# A Simple Surface-Enhanced Raman Spectroscopic Method for on-Site Screening of Tetracycline Residue in Whole Milk

**DOI:** 10.3390/s18020424

**Published:** 2018-02-01

**Authors:** Sagar Dhakal, Kuanglin Chao, Qing Huang, Moon Kim, Walter Schmidt, Jianwei Qin, C. Leigh Broadhurst

**Affiliations:** 1 Environmental Microbial and Food Safety Laboratory, Agricultural Research Service, United States Department of Agriculture, 10300 Baltimore Avenue, Bldg. 303 BARC-East, Beltsville, MD 20705, USA; sagar.dhakal@ars.usda.gov (S.D.); moon.kim@ars.usda.gov (M.K.); walter.schmidt@ars.usda.gov (W.S.); jianwei.qin@ars.usda.gov (J.Q.); leigh.broadhurst@ars.usda.gov (C.L.B.); 2 Hefei Institute of Physical Science, CAS 350 Shushanhu Road, Hefei 230031, China; huangq@ipp.ac.cn

**Keywords:** surface-enhanced Raman spectroscopy, veterinary drugs, tetracycline, milk, food safety

## Abstract

Therapeutic and subtherapeutic use of veterinary drugs has increased the risk of residue contamination in animal food products. Antibiotics such as tetracycline are used for mastitis treatment of lactating cows. Milk expressed from treated cows before the withdrawal period has elapsed may contain tetracycline residue. This study developed a simple surface-enhanced Raman spectroscopic (SERS) method for on-site screening of tetracycline residue in milk and water. Six batches of silver colloid nanoparticles were prepared for surface enhancement measurement. Milk-tetracycline and water-tetracycline solutions were prepared at seven concentration levels (1000, 500, 100, 10, 1, 0.1, and 0.01 ppm) and spiked with silver colloid nanoparticles. A 785 nm Raman spectroscopic system was used for spectral measurement. Tetracycline vibrational modes were observed at 1285, 1317 and 1632 cm^−1^ in water-tetracycline solutions and 1322 and 1621 cm^−1^ (shifted from 1317 and 1632 cm^−1^, respectively) in milk-tetracycline solutions. Tetracycline residue concentration as low as 0.01 ppm was detected in both the solutions. The peak intensities at 1285 and 1322 cm^−1^ were used to estimate the tetracycline concentrations in water and milk with correlation coefficients of 0.92 for water and 0.88 for milk. Results indicate that this SERS method is a potential tool that can be used on-site at field production for qualitative and quantitative detection of tetracycline residues.

## 1. Introduction

Tetracycline is a broad-spectrum antibiotic effective against a wide range of microorganisms including Gram-positive and Gram-negative bacteria, atypical bacteria and protozoan parasites [1]. It has been extensively used at therapeutic levels for prevention of bacterial infections in humans, animals, and plants [2], and at subtherapeutic levels in animal, poultry and fish feeds as growth promoters [3,4]. Animals receiving feed treated with antibiotics gain 4 to 5% more body weight compared to those receiving antibiotic-free feeds, which has escalated subtherapeutic use [5]. Consuming food products from tetracycline treated animals increases the risk of antibiotic resistant infections in humans if the proper withdrawal protocol is not followed. Regular consumption of tetracycline-contaminated food items can be toxic and allergenic to humans, and may induce fatty liver in pregnancy [6,7,8].

Antibiotics are frequently used for lactating dairy cows [9]. Intramammary infusion for mastitis treatment is a primary source of antibiotic contamination in milk [10,11]. Dosage in excess of label direction is fairly common in large animals, thus estimated withdrawal periods may not be adequate to ensure that antibiotic residue in milk is below the required limits [12,13,14]. The maximum residue limit (MRL) of tetracycline in milk set by the United States Food and Drug Administration is 0.3 ppm [15]. The US initiated restrictions on subtherapeutic tetracycline usage in January 2017 [16]. The tetracycline MRL of milk set by European Union and other countries is 0.1 ppm [16,17,18]. It is imperative to determine tetracycline residue levels in milk, meat and their derivative products to ensure food safety.

Several methods have been developed for detection of tetracycline residues in animal products: liquid chromatography/mass spectroscopy [8,19,20], high performance liquid chromatography [7,21,22,23], microbial tests [24,25,26,27], and electrochemical aptasensors [28,29]. Although these methods have high accuracy and low detection limits, factors including operational costs, requirement for skilled personnel, complicated sample preparation, solvent disposal and protracted sampling time limit their field application. An effective and efficient screening technique must be inexpensive, rapid, easy to operate and capable of processing multiple samples simultaneously [30]. Optical techniques such as visible/near-infrared spectroscopy, hyperspectral imaging and Raman spectroscopy are gaining importance for food safety and quality detection due to simple and inexpensive operation, minimal sample preparation and rapid results [31,32,33]. Among the optical techniques, Raman spectroscopy has the advantages of insensitivity to water, and qualitative and quantitative identification of a wide range of chemicals [34]. However, Raman spectra of biological samples are often overwhelmed by strong background fluorescence, preventing detection of chemicals at low concentrations.

Surface-enhanced Raman spectroscopy (SERS) uses metals such as gold (Au) or silver (Ag) in colloidal form to enhance Raman signal intensity, greatly lowering detection limits. SERS has been widely used in food authentication research for detection of microorganisms and contaminants [35,36,37,38,39,40] and for detection of antibiotics in biomedical and pharmaceutical applications [41,42]. SERS has been applied to qualitatively detect antibiotics in aqueous solutions, demonstrating its potential for food safety applications [43,44].

Tetracycline residues in milk should be measured immediately after milking. Prevailing laboratory-based technologies are not adaptable for on-site quantitative detection. This study used a portable Raman spectroscopic system and silver colloid nanoparticles to develop a simple method for on-site quantification of tetracycline residues in whole milk for agricultural applications. The proposed method can detect tetracycline residues in milk at concentrations below the MRL listed above. The main objectives of this study are to:

1. Develop a simple and easy to use SERS method for on-site detection of tetracycline residue in milk and water.
2. Analyze the spectra for consistently positive detection of residual tetracycline.
3. Develop a quantitative model to estimate tetracycline concentrations.

## 2. Materials and Methods

### 2.1. Portable Raman Spectroscopic System

**Figure 1:**
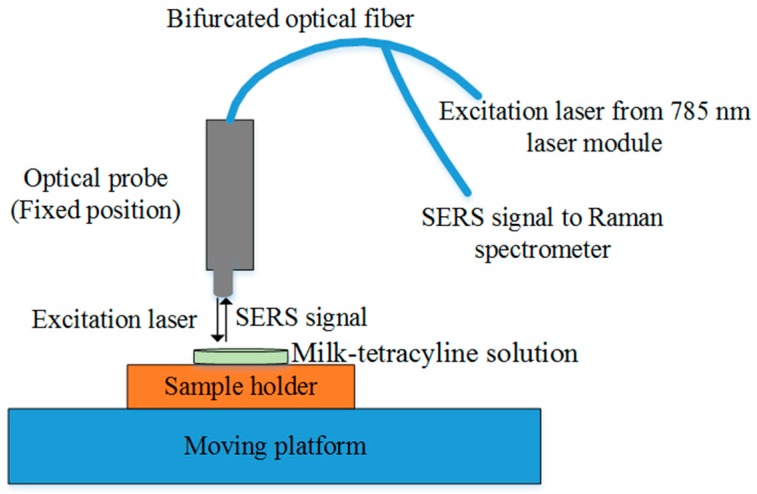
Schematic of the portable surface-enhanced Raman spectroscopic system.

Figure 1 shows the portable system used for surface-enhanced Raman spectroscopy. The Raman chemical imaging system described by Qin et al. [45] was modified. The system consists of a 16-bit charge-coupled device (CCD) camera with 1024 × 256 pixel resolution (Newton DU920N-BR-DD, Andor Technology, South Windsor, CT, USA) mounted with a Raman spectrometer (Raman Explorer 785, Headwall Photonics, Fitchburg, MA, USA). A USB cable connects the CCD camera to a computer for control and data transfer. A 785 nm laser module (I0785MM0500MF, Innovative Photonics Solutions, Monmouth Junction, NJ, USA) is used as the light source for sample excitation. A bifurcated optical fiber is connected to the laser module at one end to deliver laser light to the optical probe for sample excitation; the other end is connected to the spectrometer through a 100 µm slit to deliver scattering signal to the spectrometer. The optical probe focuses the excitation laser on the sample and collects scattering signal from the sample. A motorized platform (MAXY4009W1-S4, Velmex, Bloomfield, NY, USA) holds samples and positions them below the optical probe. Software developed in-house is used for CCD and platform control, and spectral acquisition and display. The system was spectrally calibrated using polystyrene and naphthalene. After spectral calibration, the system covered the range 111 to 2563 cm^−1^. Spectral resolution is 14 cm^−1^ at full width half maximum.

### 2.2. Silver Colloid Preparation

The method reported by Ma and Huang [46] was used to synthesis six batches of cyclodextrin capped silver colloid nanoparticles. Silver nitrate (AgNO_3_ > 99%), beta-cyclodextrin (β-CD > 97%) and sodium hydroxide (NaOH 1.0 M) (Sigma-Aldrich, St. Louis, MO, USA) were utilized without further purification. A clear solution of 50 mL deionized water and 0.4 g β-CD was prepared in a 125 mL flask and pH adjusted with 250 µL NaOH. The flask was placed in a hot water bath and heated to 80 °C, then 1.5 mL 20 mM AgNO_3_ solution was added with rigorous stirring. After 10 min, Ag colloidal nanoparticles form and the colorless solution becomes green. The nanoparticles were cooled, then purified with two cycles of deionized water wash, centrifugation (14,000 rpm/15 m) and decanting. The colloids were pipetted out and stored in clean tubes in a refrigerator.

### 2.3. Sample Preparation and Spectral Measurement

Tetracycline hydrochloride powder (>95%, Sigma-Aldrich, St. Louis, MO, USA) was mixed with deionized water to prepare stock 1000 ppm solution, then diluted to form the concentration series 500, 100, 10, 1.0, 0.1, and 0.01 ppm. Similarly, the tetracycline hydrochloride powder was mixed with organic whole milk (Costco Brand, Issaquah, WA, USA) to prepare stock 1000 ppm solution. The 1000 ppm solution was diluted by adding milk to obtain the concentration series 500, 100, 10, 1.0, 0.1, and 0.01 ppm.

One silver colloid batch was selected randomly to prepare the first set of water-tetracycline spiked samples. 5 µL 1000 ppm water-tetracycline solution and 5 µL silver colloid were mixed in a disposable aluminum dish. Three replicate samples of 1000 ppm water-tetracycline solution were prepared using this batch. Then, the same silver colloid batch was used to prepare three replicate samples of 500, 100, 10, 1.0, 0.1, and 0.01 ppm water-tetracycline solutions. The surface-enhanced Raman spectra were collected immediately after preparing the spiked samples to avoid drying. In the second week, three replicate samples of water-tetracycline solution at each of the seven concentrations were prepared in the same manner using the second silver colloid batch and SERS spectra were collected. In the third week, the third silver colloid batch was used to prepare three replicate samples of water-tetracycline solution at each of the seven concentrations and SERS spectra were collected. In this way, nine SERS spectra of water-tetracycline solution at each concentration were collected over three weeks. A total of 63 SERS spectra were collected from the water-tetracycline solution (9 replicates × 7 concentrations).

The same process was repeated using three other silver colloid batches to prepare milk-tetracycline solution spiked samples in the first, second and third week and SERS spectra were collected. Nine SERS spectra of milk-tetracycline solution—at each concentration were collected over three weeks. A total of 63 spiked samples were obtained from the milk-tetracycline solution. The spectrum of the corresponding silver colloid batch was also recorded on the day of experiment.

## 3. Results and Discussion

### 3.1. Raman Spectra of Tetracycline and Whole Milk

**Figure 2:**
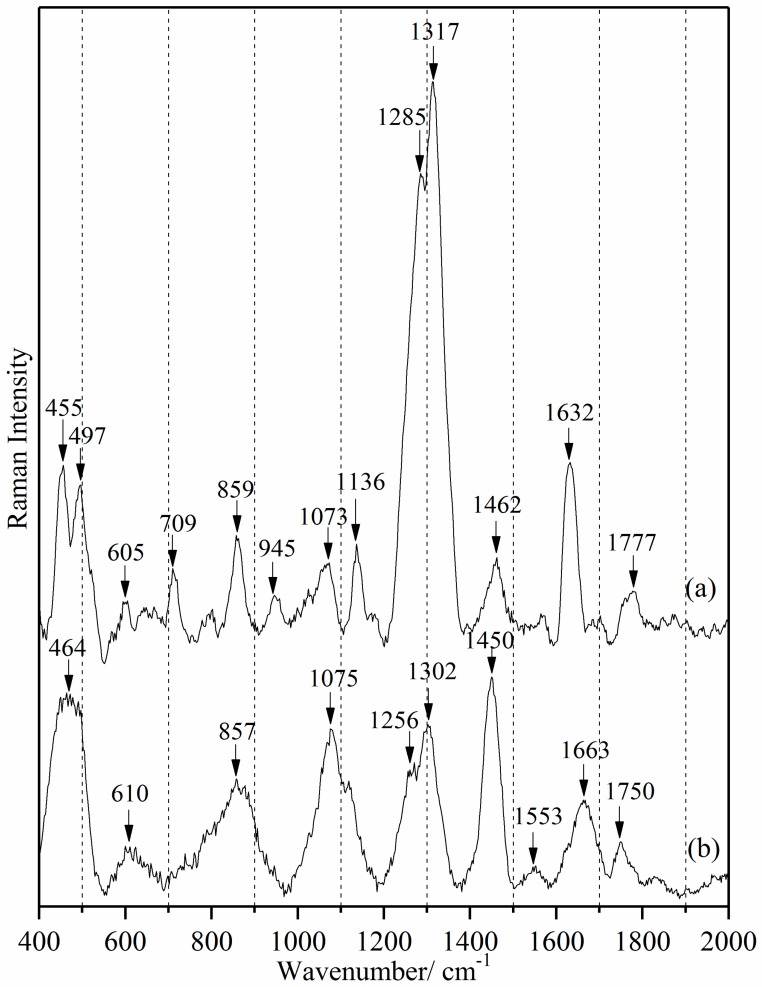
Raman spectra of tetracycline hydrochloride powder (**a**) and whole milk (**b**).

Figure 2 gives the Raman spectrum of tetracycline hydrochloride powder and whole milk. For clarity, only the range 400 to 2000 cm^−1^ is shown, but this covers all the important spectral information. The tetracycline molecular structure contains four rings (D, C, B, and A). Ring D (6a-7-8-9-10-10a-6a) is planar because of three double bonds (C7=C8, C9=C10 and C6a=C10a). The sites C9 to C12 (bottom half of molecule) are coplanar: Ring C (5a-6-6a-10a-11-11a-5a) and Ring B (4a-5-5a-11a-12-12a-4a) are planar because C6a=C10a-C11=O and O=C11-C11a=C12 are both co-planar with Ring D. The Ring A (1-2-3-4-4a-12a-1) structure is almost all above the C9 to C12 moiety plane because C12a-OH and C4a-H site are below the plane, and C3=C2-C1=O is planar and not in the C9 to C12 plane [47].

Several tetracycline peaks (605, 859, 1073 and 1136 cm^−1^) overlap with those of milk (610, 857, 1075 and 1133 cm^−1^). These milk vibrational modes correspond predominantly to proteins and lipids [48,49]. However, the tetracycline peaks at 1285, 1317 and 1632 cm^−1^ are discrete for tetracycline identification. The 1317 cm^−1^ frequency is assignable to ring breathing ν(C10-C10a), ν(C6a-C7), ν(C9-C10) relative to the planar C9 to C12 moiety. This portion of tetracycline is primarily hydrophobic and flat. The 1618 cm^−1^ frequency is assigned to ν(O-C1), ν(C2-C3), δ(amide-CO), δ(amide-NH)), i.e., amide stretching and bending modes. Ring A includes both the amide -NH_2_ moiety and secondary amine site C4-(N)(-CH_3_)_2_. Ring A thus includes the most polar and the most hydrophilic sites on the molecule. This site is clearly spatially resolved from the hydrophobic sites. The amide vibrational mode is pH dependent, so with tetracycline hydrochloride, the amide moiety would be (O=C-NH_3_)^+^. The degree of protonation of tetracycline is pH-dependent; milk is normally pH 6.6 ± 0.1 [50].

The short molecular fraction C4-C4a-C5-C5a contains only aliphatic H-C and CH_2_ sites. The 1285 cm^−1^ frequency δ(C-H) 4, 4a, 5, 5a arises from molecular sites which are the most elastic over short inter-atomic distances and these elastic sites are also localized. Frequency shifts observed in a matrix localized at only one site would be evidence for which moiety of the drug molecule is involved in tetracycline-matrix binding [51,52].

### 3.2. Raman Spectra of Cyclodextrin Capped Silver Colloids

**Figure 3:**
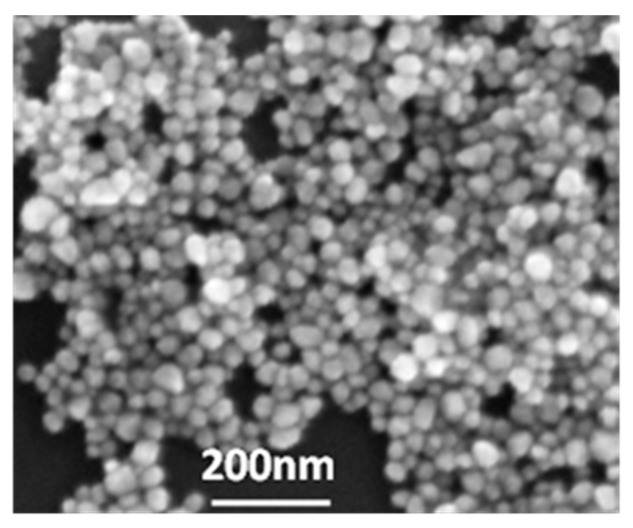
SEM image of silver colloid nanoparticles.

Figure 3 shows the silver colloid nanoparticles imaged with a Hitachi S-4800 scanning electron microscope (SEM). The nanoparticles have uniform spherical shape; diameters ranged from 30 to 50 nm, with average 40 nm.

**Figure 4:**
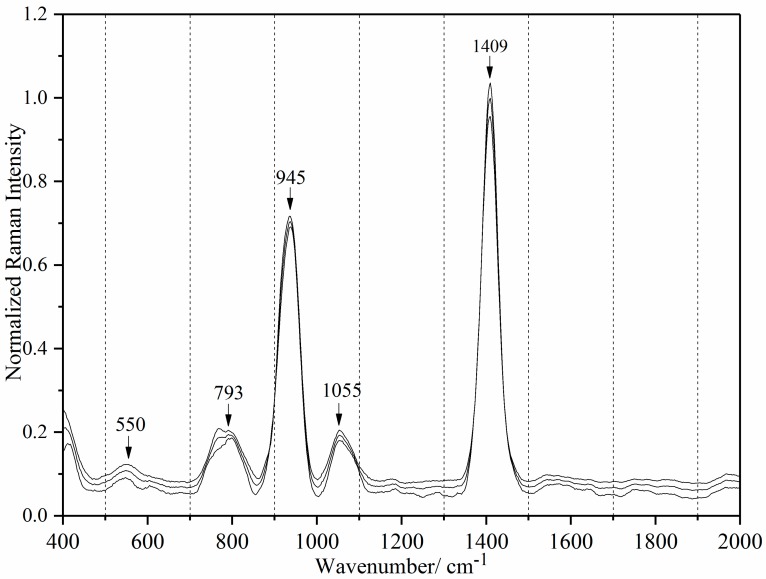
Raman spectra of mean and ±SD of six batches of silver colloids.

Ag colloid preparation is sensitive to factors including temperature, mixing, and rate of reagent addition [53,54]. Inattention to these factors results in inconsistency and irreproducibility between batches. Figure 4 shows the mean and ±standard deviation (SD) spectra of our six batches. The cyclodextrin capped Ag colloids were ideal because they produced low background signal and did not interfere with tetracycline detection. Before computing the mean, the six spectra were normalized to peak intensity at 1409 cm^−1^ = 1.0. Only five distinct peaks (550, 793, 945, 1055, 1409 cm^−1^) were observed, demonstrating reproducibility and consistency in our synthetic procedure. No significant change was observed in the spectral profile among the batches despite different storage periods. 550, 1055 and 1409 cm^−1^ are ring chair deformation, ring breathing and C7-C8 stretching + O-C8-O stretching modes on cyclodextrin, respectively [55]. 793 and 945 cm^−1^ are sites on cyclodextrin corresponding to conformational flexibility [56].

### 3.3. Surface-Enhanced Raman Spectra of Water-Tetracycline Solution

**Figure 5:**
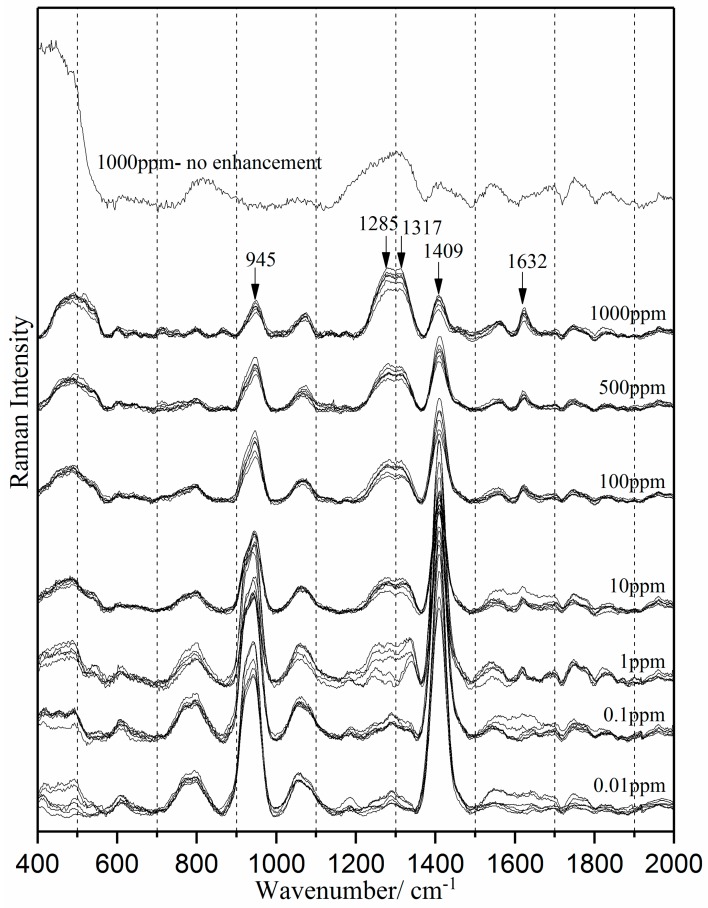
Surface-enhanced Raman spectra of water-tetracycline solution at seven concentration levels.

Figure 5 shows the SERS spectra of water-tetracycline at seven concentration levels (1000, 500, 100, 10, 1.0, 0.1, 0.01 ppm). Tetracycline peaks are present at 1285, 1317 and 1632 cm*^−^*^1^. In comparison, the 1000 ppm spectrum without enhancement (also Figure 5) contains no distinct tetracycline peaks. The increased intensity of tetracycline peaks in the SERS spectra across all concentrations demonstrates the enhancement effect of the silver colloid nanoparticles. To demonstrate consistent SERS detection across all tetracycline concentration levels, three spectra were acquired from each colloidal batch—a total of nine spectra at each concentration level. The nine spectra at each concentration level were collected over three weeks. No significant variation was observed among spectra within the same concentration level, demonstrating consistent SERS detection despite different storage periods. In Figure 5, the three predominant modes are at the same frequencies as in pure tetracycline (Figure 2a), and the 1285 and 1317 cm*^−^*^1^ peaks have a similar shape.

Tetracycline at the molecular level contains hydrophilic groups (-OH, O=C-NH, C=O, and -(N-H)(CH_3_)_2_ sites) separated by a mostly flat hydrophobic structure of four six carbon rings. Beta-cyclodextrin has an abundance of hydrophilic -OH binding sites and a 7.8Å inner diameter hydrophobic cavity [57]. The 1317 cm*^−^*^1^ intensity could be significantly lower for concentrations below 10 ppm because the tetracycline molecules absorbed inside cyclodextrin have limited space for ring breathing vibrational modes. At higher concentrations, absorption can occur on the outside of cyclodextrin as well as inside it.

The number of hydrophobic and hydrophilic adsorption sites on the silver colloid can be unequal, especially if the ratio of silver to cyclodextrin were not exactly stoichiometric, or not exactly uniform. Silver sites unbound to cyclodextrin would be hydrophilic and have a higher affinity for the amide group on tetracycline. This could explain the abrupt change in the shape and the intensity of 1632 cm*^−^*^1^ peak from 0.1 ppm and 0.01 ppm. Once the higher affinity/lower concentration hydrophilic sites are bound with tetracycline, the next binding site to be filled would be the cyclodextrin hydrophobic cavity. The 1285 cm*^−^*^1^ peak also exhibited a decreasing intensity and/or broadening at lower concentration. This peak, readily identifiable across all concentration levels, appears to be less sensitive to differences between hydrophilic and hydrophobic binding sites.

The surface-enhanced Raman spectra (Figure 5) also contains the spectral peaks at 945 cm*^−^*^1^ and 1409 cm*^−^*^1^, which are directly assigned to silver colloid. A gradual increase in 1409 cm*^−^*^1^ (cyclodextrin Ring-Chair deformation vibrational mode) peak intensities at decreasing concentration is the inverse function of tetracycline binding: maximum Ring-Chair deformation occurs in the absence of binding. Concurrent changes in the peak intensity at 945 cm*^−^*^1^ (cyclodextrin sites of elasticity vibrational modes) are consistent with an increase in cyclodextrin rigidity with an increase in tetracycline binding.

**Figure 6:**
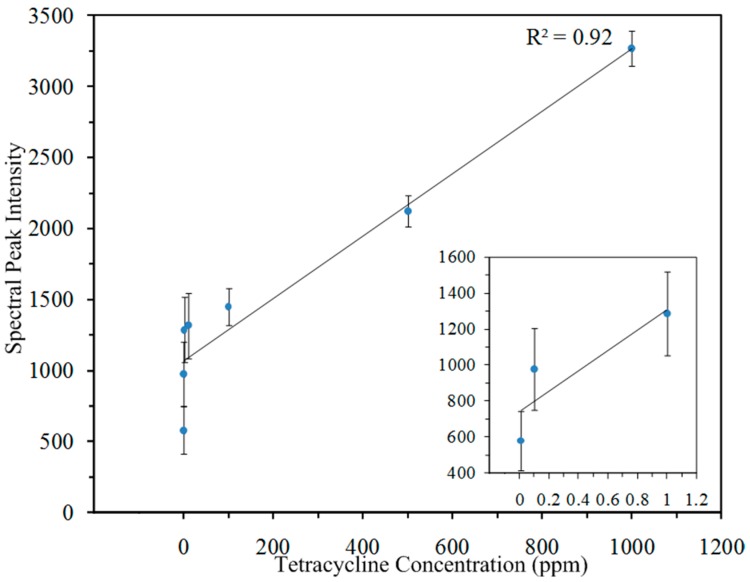
Linear relationship between tetracycline peak intensity at 1285 cm*^−^*^1^ and its concentration in water. Inset: 0.01 ppm, 0.1 ppm and 1 ppm.

The average peak intensity of the 1285 cm*^−^*^1^ mode for 9 spectra at each concentration level was used to develop a predictive model to estimate tetracycline concentrations. Figure 6 shows the linear correlation (*R*^2^ = 0.92). Note the inset shows correlations for 0.01 ppm, 0.1 ppm and 1.0 ppm. The standard deviation range is narrow at high concentration, but widens at low concentration, ranging from 111 to 226. The slopes are all similar, supporting a linear relationship between intensity and concentration.

### 3.4. Surface-Enhanced Raman Spectra of Milk-Tetracycline Solution

**Figure 7:**
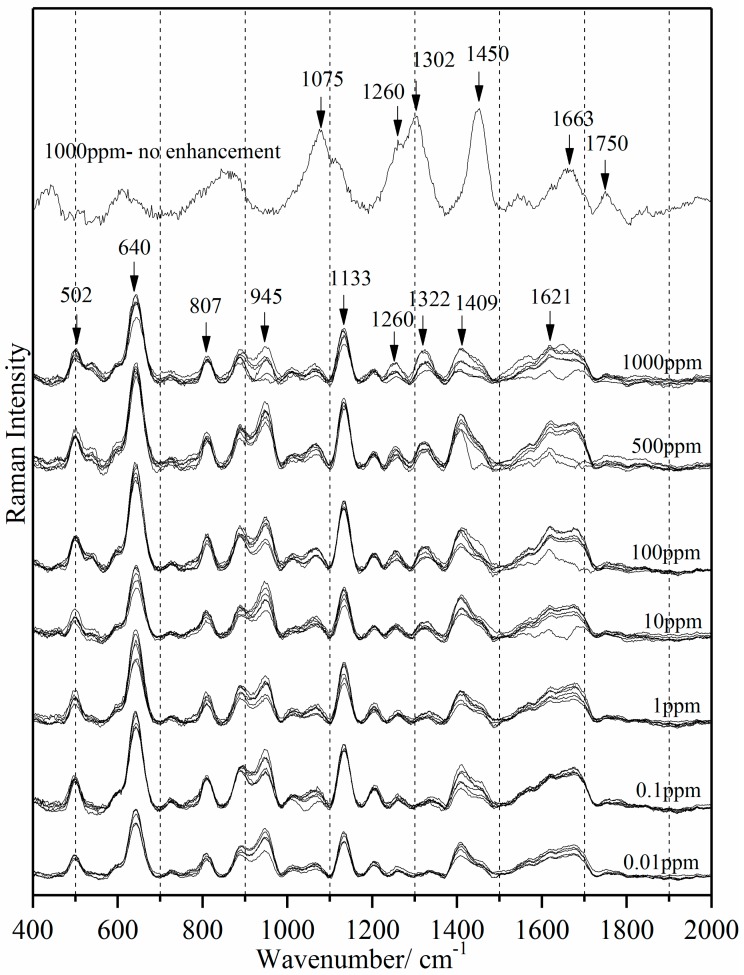
Surface-enhanced Raman spectra of milk-tetracycline solution at seven concentration levels.

Figure 7 shows the SERS spectra of milk-tetracycline at seven concentration levels and the unenhanced 1000 ppm spectrum. Only the SERS data show tetracycline peaks. Comparing the unenhanced 1000 ppm spectrum with the SERS spectra across seven concentrations, the enhanced tetracycline peaks show the surface enhancement effect of the Ag colloid nanoparticles. The 1322 and 1621 cm*^−^*^1^ peaks (shifted from 1317 and 1632 cm*^−^*^1^ respectively) are attributed to tetracycline. These frequency shifts indicate that tetracycline is included into a beta-cyclodextrin moiety. Comparing Figure 2a, milk-tetracycline solution has an overlap of 1285 cm*^−^*^1^ with milk at 1256 cm*^−^*^1^ to form a broader 1260 cm*^−^*^1^ peak. Additionally, 1265 cm*^−^*^1^ is a lipid peak, corresponding to CH_2_ twisting in the double bond region of linoleic acid [58]. Thus, tetracycline may also be binding with any polyunsaturated fats present in milk fat.

No significant change in the spectral profile and the position of the SERS tetracycline peaks were observed in nine spectra within the same concentration level in Figure 7, confirming the consistent SERS detection across all tetracycline concentration levels. The consistent SERS detection of tetracycline shows the stability of silver colloid nanoparticles when used with milk. This also confirms that storage of the silver colloids for three weeks does not influence SERS tetracycline detection.

The milk peaks (such as 640 and 1133 cm*^−^*^1^) and silver colloid peaks (945 cm^-1^ and 1409 cm*^−^*^1^) exhibited no distinct variation in the peak intensity across the seven concentration levels. Two possible reasons can explain the lack of change in peak intensity of the silver colloid in milk solution (in contrast to water solution, which exhibits increasing intensity with decreasing tetracycline concentration). First, the pH of milk is naturally strongly buffered so the concentration of hydrophilic sites in milk remains quite constant. In water, the pH can change with increasing (though low) concentrations of tetracycline HCl. Second, the cyclodextrin binding cavity could be more stable and/or a better fit for adsorption in a more hydrophobic environment.

**Figure 8:**
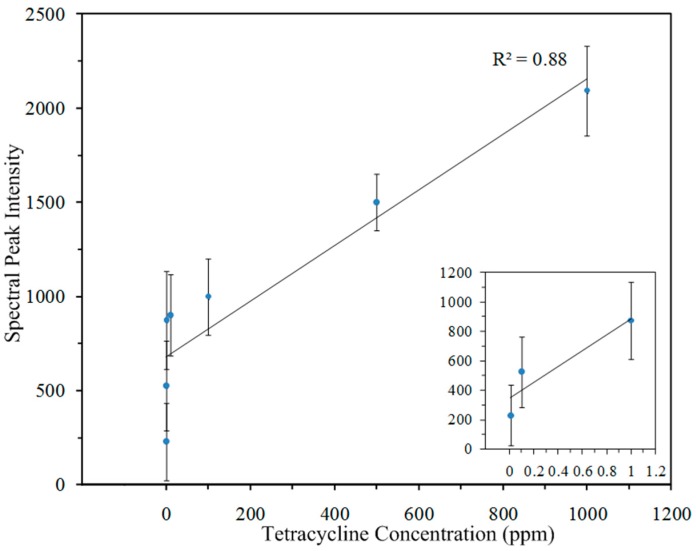
Linear relationship between tetracycline peak intensity at 1322 cm^−1^ and its concentration in milk. Inset: 0.01 ppm, 0.1 ppm and 1 ppm.

The peak intensities at 1322 and 1621 cm*^−^*^1^ were attenuated with decreasing concentration. The 1621 cm*^−^*^1^ peak is not apparent below 0.1 ppm concentration, but the tetracycline peak at 1322 cm*^−^*^1^ is detectable to 0.01 ppm. The intensity of the 1322 cm*^−^*^1^ peak was correlated with the actual concentration of tetracycline in milk solution. The average peak intensity of 9 spectra at each concentration level and the standard deviation was computed. Figure 8 shows the linear correlation between the 1322 cm*^−^*^1^ intensity and tetracycline concentration (*R*^2^ = 0.88).

## 4. Conclusions

This study demonstrates a simple, on-site SERS method for detection of tetracycline residue in bovine milk. A customized portable 785 nm Raman spectroscopic system coupled with silver colloid nanoparticles was used to detect tetracycline in milk and deionized water at concentrations 1000, 500, 100, 10, 1.0, 0.1, and 0.01 ppm. The reproducibility and consistency of silver colloids was assessed by producing six batches with matching spectral peak positions.

The characteristic vibrational modes of tetracycline at 1285, 1317 and 1632 cm^−1^ in water-tetracycline solution and 1322 and 1621 cm^−1^ in milk-tetracycline solution consistently appeared after surface-enhancement, but were absent in unenhanced spectra. The peaks at 1317 cm^−1^ and 1322 cm^−1^ can detect tetracycline in water-tetracycline solutions and milk-tetracycline solutions at concentrations as low as 0.01 ppm, which is below the MRL set by government authorities. The peak intensities of 1317 and 1322 cm^−1^ were linearly correlated to the concentrations with correlation coefficients of 0.92 for water and 0.88 for milk. The method developed in this study can field-detect residual concentrations of tetracycline in milk and water. Results serve as a foundation to adopt the method for further research to detect other possible veterinary drugs in animal products.
